# Sea lions could use multilateration localization for object tracking as tested with bio-inspired whisker arrays

**DOI:** 10.1038/s41598-022-15904-1

**Published:** 2022-07-11

**Authors:** Raphael Glick, Muthukumar Muthuramalingam, Christoph Brücker

**Affiliations:** School of Mathematics Computer Science and Engineering, City University of London, London, EC1V 0HB UK

**Keywords:** Engineering, Biomimetics

## Abstract

Previous behavioural research on live sea lions has shown that they are able to detect the direction of oncoming vortices, even when impacting contralaterally. These experiments showed that the whisker system and the animal’s neural processing is seemingly able to detect the Direction of Arrival (DoA) from just one side of the heads vibrissal pads. Therefore, temporal differences between whisker stimulation is a likely method for determining the angle. Herein, a theoretical model is presented based on multilateration, and tested by experimental studies on a 2D array of bio-inspired whiskers with regular spacing, and a 3D array of bio-inspired whiskers on a model head of a sea lion, as used in our previous studies. The results show that arrays of whiskers can in principle work as antennae to determine the DoA. This detection of the DoA is achieved by cross-correlation of triplets of whiskers, and Time Difference Of Arrival based multilateration, a method similar to signal processing in modern communication systems and other source localization applications. The results on the 2D array are conclusive and clearly support the hypothesis, while increased uncertainties were found for the 3D array, which could be explained by structural shortcomings of the experimental model. Possible ways to improve the signal are discussed.

## Introduction

The capability of sea lions to track prey-induced flow disturbances in the aquatic environment, even long after the source has passed the path of the sea lion, is still a fascinating subject of research. It shows the extraordinary sensitivity of the natural whiskers and the neural processing, which allows the sea lion to distinguish between different sources and the specific pattern left in their wake. Bio-inspired whisker sensing systems, with high sensitivity and selectivity, are highly useful to autonomous underwater vehicles, and would expand upon existing sensing capabilities in diverse aquatic environments. Increasing concern around the environmental impact of active sonar^1,2^ make such systems unappealing for some applications, creating demand for alternative, more unobtrusive systems, that can detect a range of different signals. Artificial Whisker-like flow sensors are being tested with the goal of extracting information from the flow, either in form of vibrational response or bending response. On a small scale, micro-pillar sensor in form of small cantilever beams have been used to study near-wall flow features optically^3^. Other research groups have tested MEMS-based electronic stress sensors attached to the base of whisker like sensors for various applications, across a wide range of sizes^4,5^. Other recent technologies utilising similar sensing principles include patterned carbon nanotube and silver nanoparticle composite films^6^ which use the piezoelectric effect to map the stress at the base of cantilever beams. These systems provide a basis for further research on hypotheses of fluid-structure interaction of arrays of sensors, and to better understand the principles of multi-sensor signal processing in the neural system of the sea lion, something which can otherwise be difficult to gain from live seal experiments.

Detailed flow-structure interaction was mostly tested first on single artificial whiskers, where the source of flow disturbance was often imposed in form of a von-Karman vortex street, simulating the wake of fish and generated by placing a cylinder upstream of the whisker. This causes a disturbance pattern that contains quasi-periodic fluctuations, as produced by the passage of rows of vortices, produced by the perpetual shedding from the shedder body. Consequently, the focus of research was to investigate the adaptation of the sensing system to detect dominant frequencies within an otherwise unidirectional mean flow. As such, the hypothesis of Galloping adaptation^7^ evolved from such experiments. The results are based on an ideal situation when the hydrodynamic signal is a regular chain of closely staggered tubular vortex structures, aligned perpendicular to the planar whiskers. Another hypothesis, the jerk-type response adaptation, was developed based on similar studies with von-Karman street type hydrodynamic disturbances using a sea lion head model, developed in our lab. Studies with arrays of artificial whiskers have shown that deep neural network systems are able to predict the location of the source of the von Karman wake (produced by a cylinder in the flow) in the near field with good accuracy in both distance and angle of the source relative to the snout^8^. Those measurements were based on the rms-values of the whisker tip fluctuations as a measure of the fluctuating bending stress at the root. Good predictability was achieved when the system was trained with the same source in repeated positions. Additionally, the wake passage was always parallel to the body axis. An open question remains as to how such a system will respond to single events that hit the whiskers from different directions or with different strengths. In nature, the hydrodynamic trail of an upstream swimming object could also consist of rather isolated vortex structures, such as for a burst and glide swimming style^9^. Therefore, the signal will not always be periodic, and the flow disturbances could have both unknown speeds and directions of origin.

Recent animal studies from the University of Rostock, Germany^10^, have proven that the sea lion is reliably able to detect the direction of arrival of a single disturbance in form of a vortex ring, shot at the seal’s head from some distance away. These results were obtained from behavioural studies of blindfolded stationary harbour seals, which were trained to respond to single vortex rings. The results show that harbour seals are able to correctly identify a variety of different vortex rings by turning their head towards the source, upon vibrissal stimulation. The authors speculated from their results, that a possible way that sea lions determine the direction of the vortices, is via the combined stimulation of the vibrissae of both vibrissal pads (left and right in the lateral plane) with a velocity difference. This would be comparable to the intensity difference in auditory localisation^11^. Indeed, the results obtained with the bio-inspired sea lion head model^8^ have shown, that for periodic excitation by vortices in a von-Karman wake, it is possible to detect the direction of the source just from the magnitude of the whiskers vortex-induced vibrations (VIV) when compared within the array. This hints to a mechanism which is sufficiently predictive just via left-right comparison of the intensity of excitation. However, one striking result of the Rostock’s group studies was that seals can also be trained to detect the travel direction with contralateral stimulation. This means that the vortex ring was passing only one single pad and without direct impact on the muzzle, see travel path 3 and 4 in Fig. 2, of their work^10^. This is the more natural situation when the sea lion encounters the wake of a swimming object with the whiskers protracted. The results showed that the animal was able to turn their head towards the direction of the source, on the opposite side to where the disturbance had been received. Therefore, it is important to further research the influence of temporal coding within the array on the response. Interesting in that context is the recent study^12^ with pairs of artificial whisker proving that a pair-wise cross-correlation of whiskers can decode the convection velocity. These time delays can be further used to triangulate the position of the source, using techniques adapted from radio localisation. The present study expands on this work and uses Cross Correlation (CC) to generate Time Difference of Arrival (TDOA) matrices for the responses of multiple sensors in a single multi-sensor array. The time delay estimates are provided here by calculating the cross-correlation between the whisker signals and searching for the time-lag that maximizes it. Multilateration of the time delays then provides the DoA.

## Methods

### Vortex ring generator

**Figure 1:**
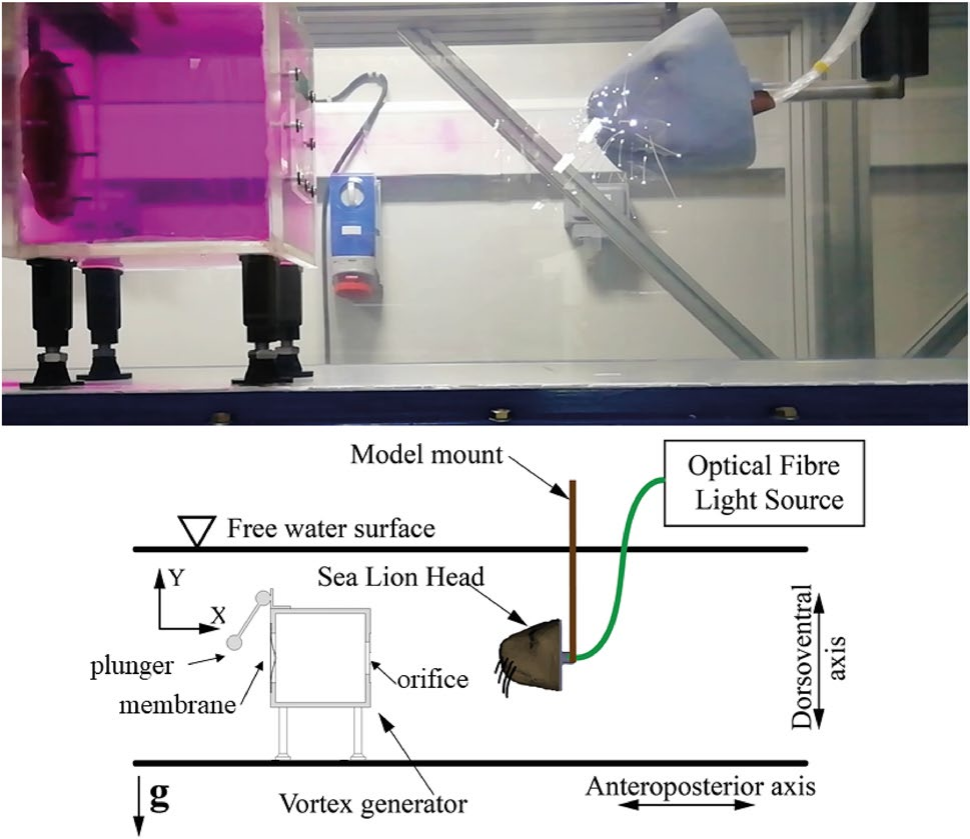
Top: Picture of the experimental setup showing the vortex ring generator filled with pink dye, 200 mm away from the model of the sea lion head with its 3D array of whiskers. Bottom: principle sketch of the experiment, including the definition of the body axes.

**Figure 2:**
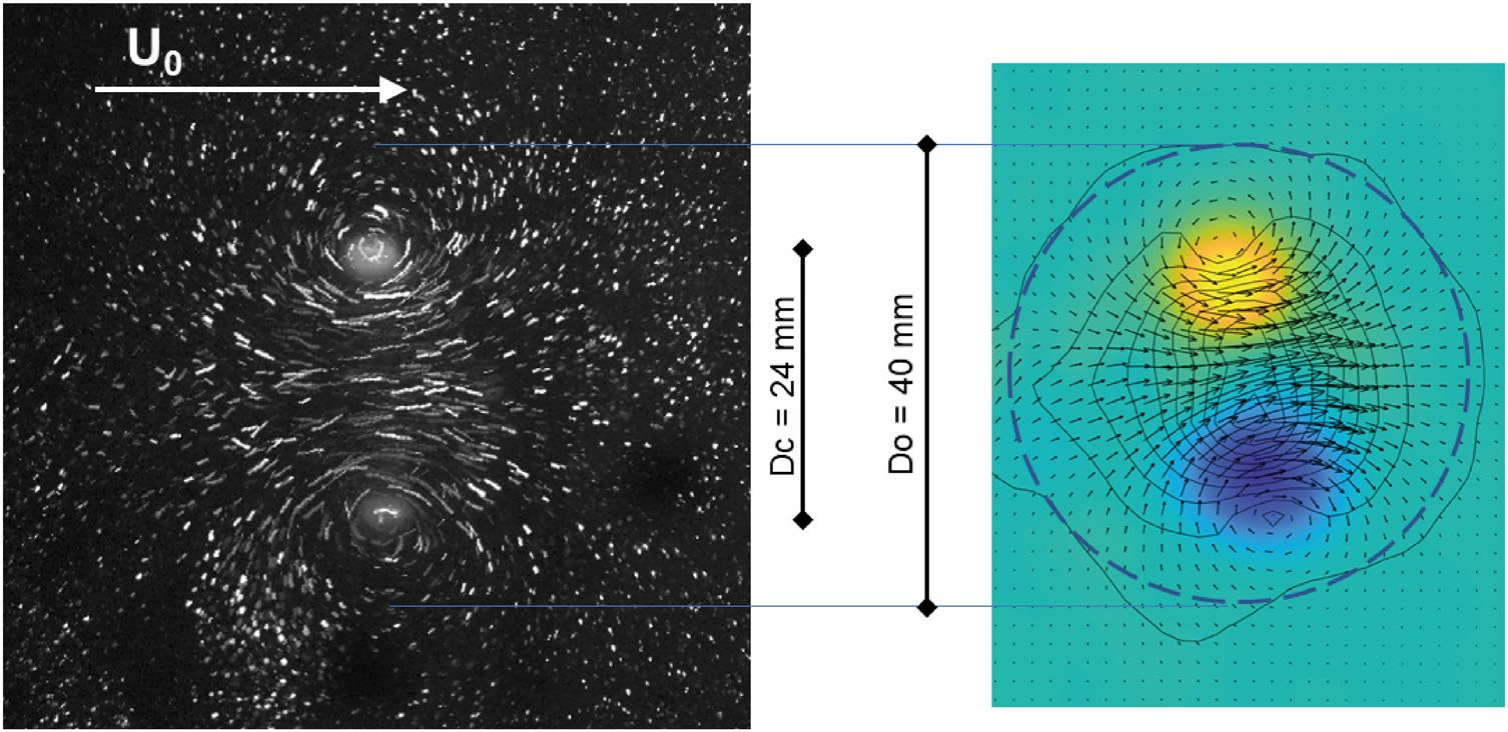
Left: Multiple exposure visualisation of the vortex ring in the horizontal plane. Right: In-plane velocity vector field induced by the travelling vortex ring. The additional contour lines show the velocity magnitude and start from 1/10 $U_0$ in steps of 1/5 $U_0$, where $U_0$ is the travel speed of the vortex ring. The color-coded vorticity field highlights the cores as regions of highest magnitude of vorticity, which represent the cuts through the toroidal core of diameter $D_c$ = 24 mm. The dashed circular ring approximates the outer diameter $D_o$ of the ring, beyond which fluid has remained nearly stagnant with velocities lower than 1/10 $U_0$.

The vortices tracked in this study were generated using a simple vortex canon (side length 200mm), modelled on the vortex canon used in the Rostock study on live sea lions^10^, designed to mimic disturbances typical of swimming prey^13^. A spring loaded plunger as shown in Fig. 1 strikes the elastic membrane on the back, which then transfers the momentum into the formation of a vortex ring at the circular opening at the opposite site. The vortex generator is installed in a water channel with free surface, used here as a simple water tank with no flow. Each push with the plunger on the membrane creates a vortex ring (outer diameter Do = 40mm, toroidal core diameter Dc = 24 mm see Fig. 2), travelling towards the model head at a speed of roughly $U_0$ = 1.1 ± 0.1 m/s, calculated using high speed footage of a dyed vortex ring fired along a ruler The speed drops slightly across the length of the model due to the interaction with the displacement body. Because of the short distance between the array tips (< 0.05 m) it is considered to be constant along this scale. A visual impression of the vortex ring is obtained from food dye, which is added to the water within the cavity of the vortex canon and is entrained into the vortex ring during generation. In addition, for further visualization of the flow pattern around the travelling vortex, small tracer particles (Potters Industries, conduct-O-Fil silver-coated ceramics, diameter 80micron, PA, USA) were added to the water along the expected path of the ring. A typical picture of the disturbance flow pattern in the otherwise quiescent environment is given in Fig. 2 by means of a multi-exposed image of those tracer particles when illuminated with a light-sheet (Hardsoft, PL, IL-105/6X Illuminator). The flow field can be processed by the method of Particle-Image-Velocimetry, for further details see our previous paper^14^.

**Figure 3:**
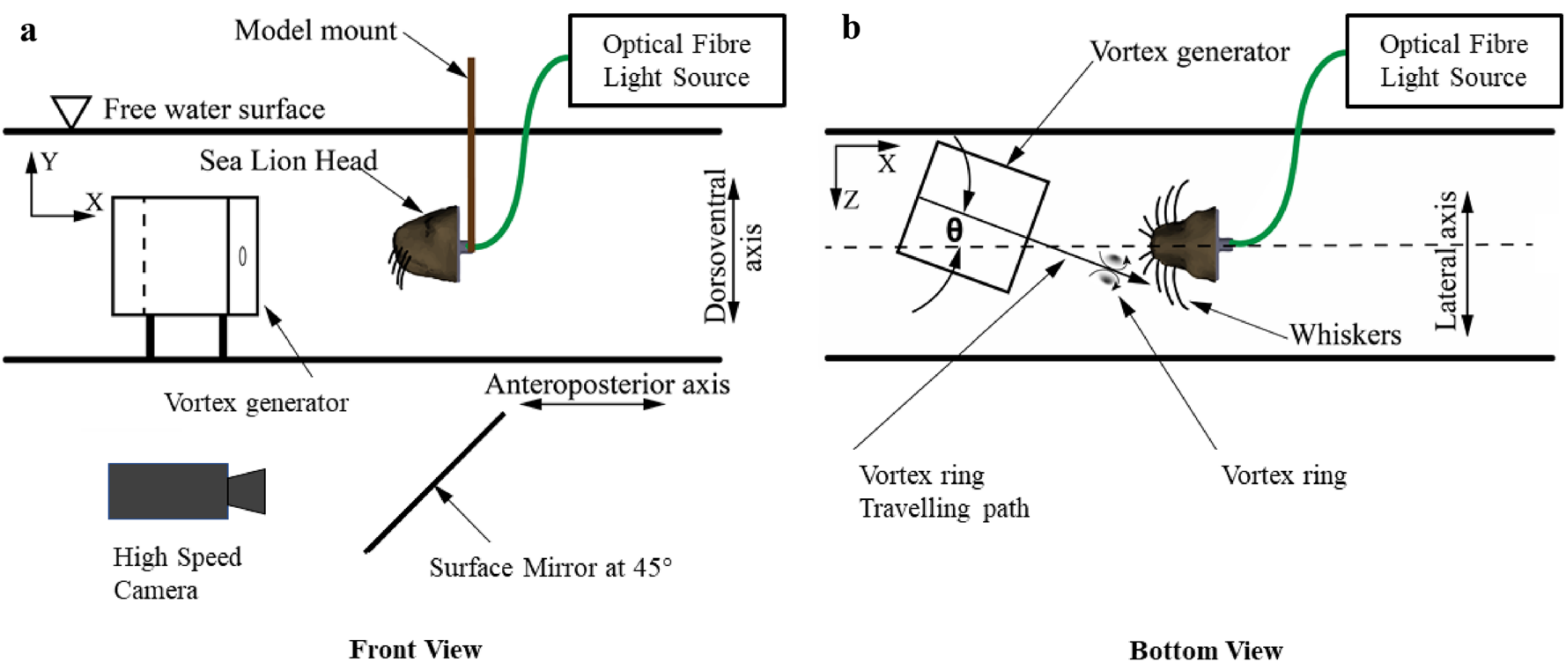
Schematic diagram of the seal head experimental set-up. To test different contralateral angles of impact, the vortex cannon is rotated around the head in between measurements. $\theta =$ 0$^\circ$–45$^\circ$.

For the 3D array studies, the vortex generator is rotated around the model in the horizontal plane to simulate vortices arriving from various angles, testing the contralateral interaction with the sea lion model as depicted in Fig. 3. For the 2D array experiments the vortex ring generator is kept stationary, but instead the model, placed in the same position as the seal head, is rotated around its vertical axis to simulate the different angles of arrival to the vortex generator.

### Sea lion model with 3D whisker array

This experimental model is the same as described in our previous work^8,14^ and the key features are described herein again. We use a 1:1 scaled model of the head of a sea lion with artificial whiskers (optical fibres with circular cross-section of diameter $d\,=\,0.75$ mm). The fibres are placed along the head with their origin at similar positions to the biological example and point outwards with a similar length variation. In addition, the material of the fibres has an elastic modulus, and diameter, similar to the natural whiskers. The array of optical fibres are inserted through holes from the backside of the 3D printed model and are illuminated from one end by an LED source such that the tips of the free fibre ends appear as bright spots, on both sides of the head. The fibres therefore represent one-sided clamped flexible cantilever beams with circular cross-section, interacting with the flow.

### Planar surface with 2D regular whisker array

**Figure 4:**
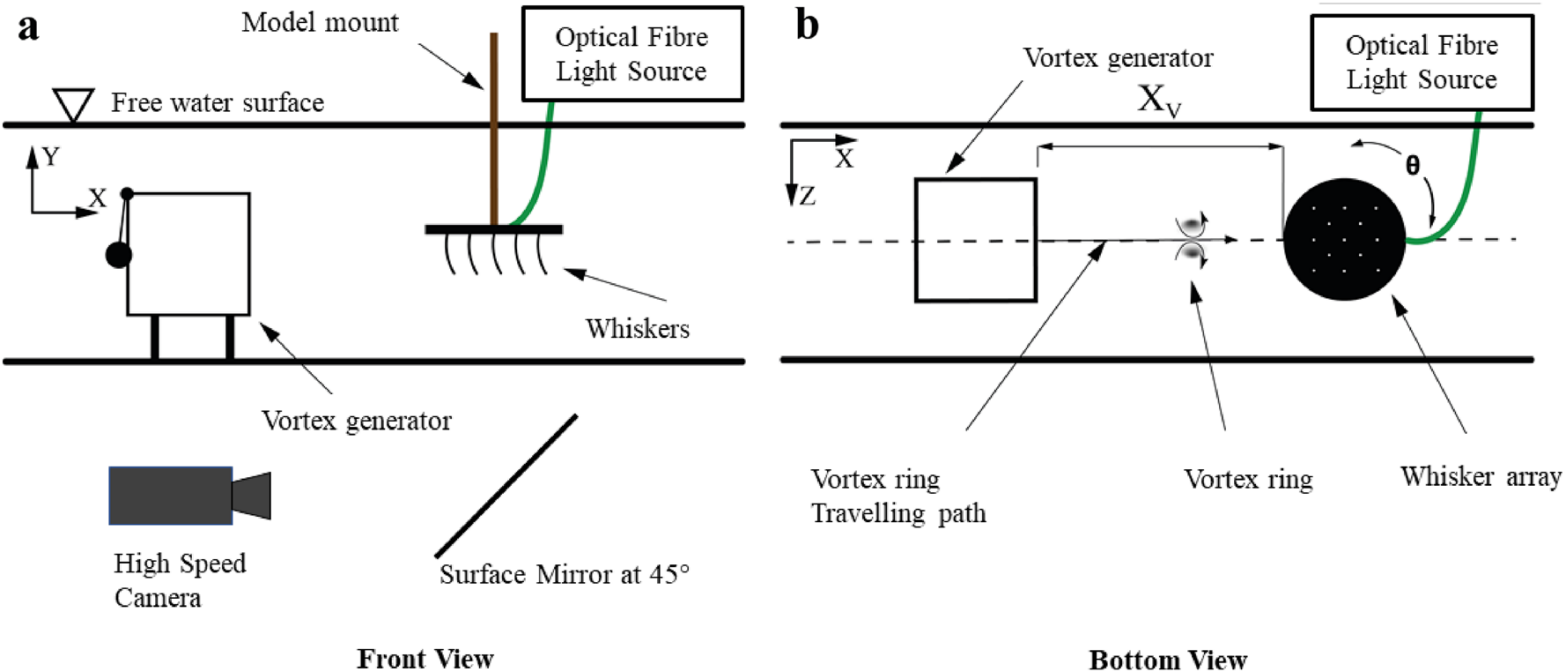
Schematic diagram of the 2D whisker array experimental set-up. To test different angles of impact within the array, the round disc housing the whisker array is rotated around its vertical axis. $\theta =\, \pm \,45^\circ$.

Another experiment is done with the same type of optical fibres arranged now in a regular array to provide reference data for a more simplified situation (compared to the artificial sea lion head). Therefore, a flat disc is used in horizontal alignment, which has clamped 13 optical fibres of a length of 100mm, pointing downwards along their vertical axes. The artificial whiskers are all arranged parallel to each other within a regular Cartesian grid pattern with a side length of 40 mm between two fibres and with the tips all lying in the same plane. The water tunnel and vortex generator are arranged as shown in Figs. 4, and 7. The interspacing between the fibres is chosen such that the circular-shaped disturbance pattern induced by the vortex ring (see Fig. 2) is always overlapping an area of minimum 3 whiskers simultaneous at each location within the grid. The disc with the fibres can be freely rotated on its mount around the vertical axis.

### Experimental setup for motion tracking

The tips of the optical fibres were tracked using a high-speed camera (ProcImage 500-Eagle high-speed camera) at a sampling rate of 250 Hz, as shown in Fig. 3. It has a pixel resolution of 1280 $\times$ 1024 which relates to physical dimensions of 221 $\times$ 177 mm. The built-in function in the camera calculates the Cartesian tip coordinates of each light spot automatically using the barycentre mode. The measured signals are the magnitude of tip deflections (Euclidian norm) of each of the optical fibres captured simultaneously, which is directly proportional to the applied bending moment at the shaft of the whiskers (Euler–Bernoulli beam theory)^8,14^. This system was used to track the sensors on both of the models. Note that the camera viewing position ensures the capture of the major components of the tip displacements, but for the 3D array the optical tracking of the tips only can capture the projection of the displacement vector, which is in the direction of the travelling vortex. A full description of the displacement vector in all directions would require an additional camera looking from another viewing direction in a stereo-camera arrangement, for which an additional camera was not available.

The maximum variation in the tracking software’s reported position of an undisturbed sensor tip due to background noise is 0.5 px, which is very low. This stems from uncertainties in the tracking code and scattered light. When this noise is compared to the smallest tip deflections on signal arrival, measured at roughly 15 px, this still gives a high Signal to Noise Ratio (SNR) of at least 30.

### TDOA from generalised cross correlation (GCC)

Using the generalized cross-correlation (GCC) algorithm, or derivatives thereof, is a widely used method for the TDOA estimation^15,16^. Here we consider a pair of whiskers being disturbed by a hydrodynamic signal.

The signals used in the GCC of each pair of whiskers are defined as:

1 $$\begin{aligned}&W_1(t) = {s}(t) + n_1(t)\nonumber \\&W_2(t) = {s}(t+D) + n_2(t) \end{aligned}$$

where W denotes a whisker, s is the recorded signal given off by the source, D is the time delay, and n is random, uncorrelated, measurement noise. The sources of measurement noise present are scattered light from the fibre tips and the uncertainty in the tracking camera’s centering predictions. Therefore, for each whisker the signal is the recorded position of the whisker tips.

The true value of the GCC is obtained via^17^:

2 $$\begin{aligned} R_{w1w2}^{(g)}(\tau ) = \int _{-\infty }^{\infty }\psi _g(f)G_{x_1x_2}(f)e^{j2\pi f\tau }df \end{aligned}$$

where $\psi _g(f)$ is the general frequency weighting, and $G_{x_1x_2}(f)$ is an infinite observation of the signal. Hence the GCC between $W_1$ and $W_2$ for a finite signal ‘t’, is estimated as:

3 $$\begin{aligned} {\hat{R}}_{w1w2}^{(g)}(\tau ) = \int _{1}^{t_{max}}\psi _g(f){\hat{G}}_{x_1x_2}(f)e^{j2\pi f\tau }df \end{aligned}$$

where ${\hat{R}}$ represents an estimate of the GCC when R is evaluated for the finite series of $x_{1,2}(t)$.

The maximum value of ${\hat{R}}$ corresponds to the delay, D, or TDOA, between each whisker pair. These calculations are repeated for each whisker pair, and therefore this GCC function outputs the set of time delays between every pair of whiskers in the array.

### DoA calculations in 2D

The Direction of Arrival is the defined as the angle between the oncoming vortex ring and the anteroposterior axis of the model, in the horizontal plane. The bow-wake effect of the travelling vortex ring is causing the initial deflection of the whiskers and is approximated across small distances as a signal propagating with a planar wavefront. This approximation is well supported by PIV measurements of the vortex ring. Figure 2 shows that the fastest moving parts of the flow form a rough line across the front of the ring.

**Figure 5:**
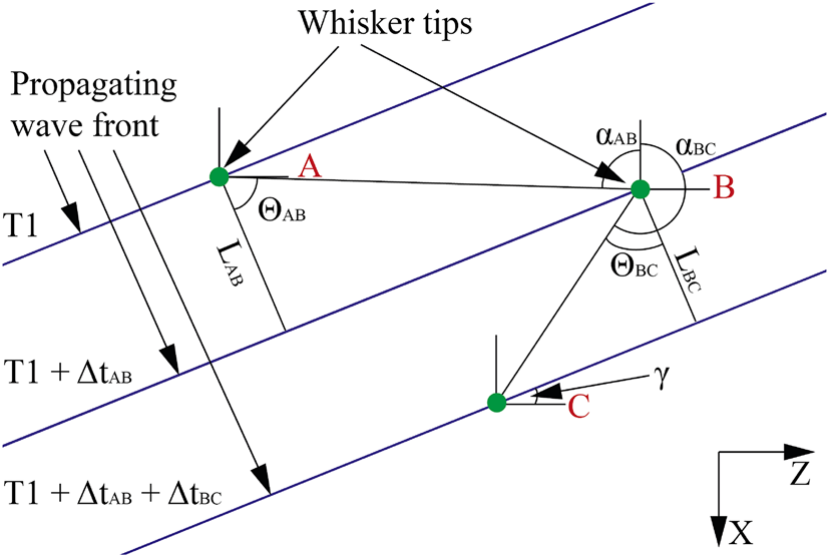
An example triplet of whisker tip points, showing how TDOA multilateration calculations are adapted for this purpose. The blue lines represent the position of the bow wave front, as it passes by and disturbs the sensors.

The inputs to the multilateration function are the arrays of time delays between every pair of whiskers, and the X and Y position of each whisker tip while the whisker is at rest. The time delays are calculated via the aforementioned GCC, as a function of the whisker's displacement (see above).

The calculation relies on the basic principle of TDOA multilateration. The following data processing is similar to passive sonar or other audio localisation microphones^18^. It differs from these conventional usage cases in that both the time of the signal's emission, and the speed of the signal are unknown. To account for this, herein triplets of sensors are used instead of the conventional pairs^19^, used when the speed of the signal, or the signal emission time are given. This allows for the calculation of the DoA by comparing the ratio of the TDOA with the ratio of the distance between the sensors, a comparison that is independent of the signal’s velocity or time of emission.

Assuming that the direction and velocity of the signal remain constant when traveling over the triplets, looking at the time difference between the excitation of three whiskers of known positions should produce the angle and velocity of the signal as follows (notation follows Fig. 5):

4 $$\begin{aligned} \frac{\Delta T_{AB}}{\Delta T_{BC}} = \frac{L_{AB}(\gamma )}{L_{BC}(\gamma )} \end{aligned}$$

where ‘$\Delta$T’ is the time difference between the whiskers A and B, and ‘L’ is the distance between points A and B in the direction of travel.

5 $$\begin{aligned} L_{AB}(\gamma ) = cos(\Theta _{AB})\sqrt{(x_{B}-x_{A})^2+(z_{B}-z_{A})^2} \end{aligned}$$

The coordinates ‘x’ and ‘z’ represent the pixel positions of whisker tips A and B, and $\Theta _{AB}$ is the angle between $L_{AB}$ and the line AB:

6 $$\begin{aligned} \Theta _{AB} = 180 - \gamma - \alpha _{AB} \end{aligned}$$

where ‘$\alpha _{AB}$’ is the angle between the distance vector $\mathbf {AB}$ and the z-axis, and ‘$\gamma$’ is the angle of the propagating signal front.

To find the solution, an algorithm is applied that scans all possible values of $\gamma$, from $- \, 90^\circ$ to 90$^\circ$ in 0.002$^\circ$ increments, recording the range of values of $\gamma$ that satisfy equation 4 to four significant figures. The size of the range of resulting values provides an estimate of the uncertainty in the angle output of that whisker triplet, and is mostly dominated by the size of $\gamma$, as small angles result in small time delays between whiskers in the triplet, which are therefore more sensitive to random error. This is repeated for all possible triplets of whiskers, and the result is taken from the displacement-weighted average of the $\gamma$ outputs:

7 $$\begin{aligned} {\bar{\gamma }} = \frac{\sum _{n=1}^{m}(\gamma _{m} * \frac{MinD}{{\bar{D}}})}{m} \end{aligned}$$

where ‘MinD’ is the minimum displacement of any whisker in the triplet, and ‘${\bar{D}}$’ is the average whisker displacement across all measurements.

The travel velocity of the signal is therefore simply estimated as:

8 $$\begin{aligned} {\hat{V}}_s = \frac{\sum _{n=2}^{p}\frac{L_{1,n}({\bar{\gamma }})}{\Delta T_{1,n}}}{p-1} \end{aligned}$$

where $L_{1,n}({\bar{\gamma }})$ is the length between points 1 and n, at the calculated ${\bar{\gamma }}$, and $\Delta T_{1,n}$ is the corresponding time delay. Error in the travel velocity due to recorded vertical variation in vortex exit angles ($\pm 2.7^\circ$) is calculated as follows:

9 $$\begin{aligned} {\%Error = \frac{{\hat{V}}_s - {\hat{V}}_s*Cos(2.7^{\circ })}{{\hat{V}}_s*Cos(2.7^{\circ })}*100} \end{aligned}$$

Lowest recorded velocities were roughly 1 m/s, leading to a maximum error of 0.11%.

## Results

**Figure 6:**
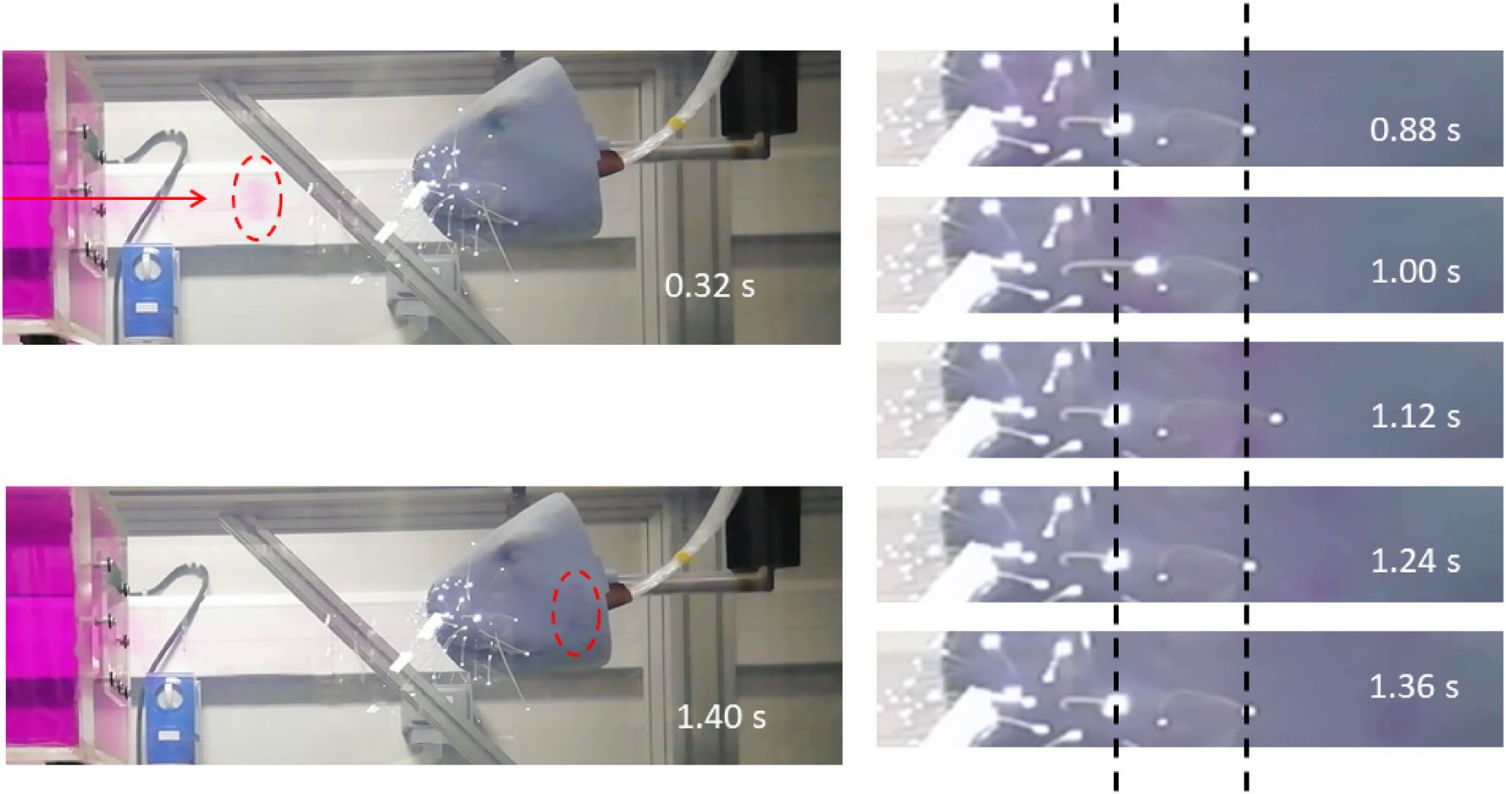
Picture of the 3D printed sea lion head with integrated optical whiskers, seen by the bright spots at the tips. The time series on the right-hand side with zoom-in illustrates the successive excitation of two neighbouring whiskers in the same horizontal plane when hit by the travelling vortex ring (travel direction from left to right).

Figure 6 shows the interaction of the vortex ring with a tandem pair of whiskers in the 3D array, which are both roughly arranged in the horizontal plane and in line with the horizontal travel direction of the vortex. For simplicity when dealing with the sea lion model, only the interaction along the head in the horizontal plane crossing the mid section of the head is tested, while the angle of impact is varied in this 2D plane. Only the whiskers which are near to each other, along the path of the vortex, and have similar length, are used for the source localisation. The time-series of the spots of the whisker tips clearly highlights the response to the bow wake of the incoming vortex ring. The leading whiskers reacts first at 1 s after the pulse initiation and the trailing one reacts 0.12 s later with a longitudinal shift of the same order as the leading one. This time-difference demonstrates the feasibility of using the tip displacement signal to determine the TDOA in the pair of whiskers. The two whiskers spotted in the figure are part of a triplet which is used to calculate the DoA with the above method. Compared to our previous studies described in Muthukumar^14^, there is no clear jerk-type motion of whiskers during the passing of a vortex. Here, the momentum of the fired vortex rings dominates the whiskers’ motion by flow-induced drag in an otherwise stationary flow field. This is different to the situation when vortices are convected in an established flow past the whisker as in the wake of a cylinder used in Muthukumar^14^, where the whiskers show a stronger response to the pressure field. Additionally, the vortex ring’s induced pressure field has a toroidal shaped core of low pressure, while the von-Karman vortices generated from a cylinder have a rather tubular axial core of low pressure. This aspect influences the interaction of the filamentous whiskers with the pressure field.

**Figure 7:**
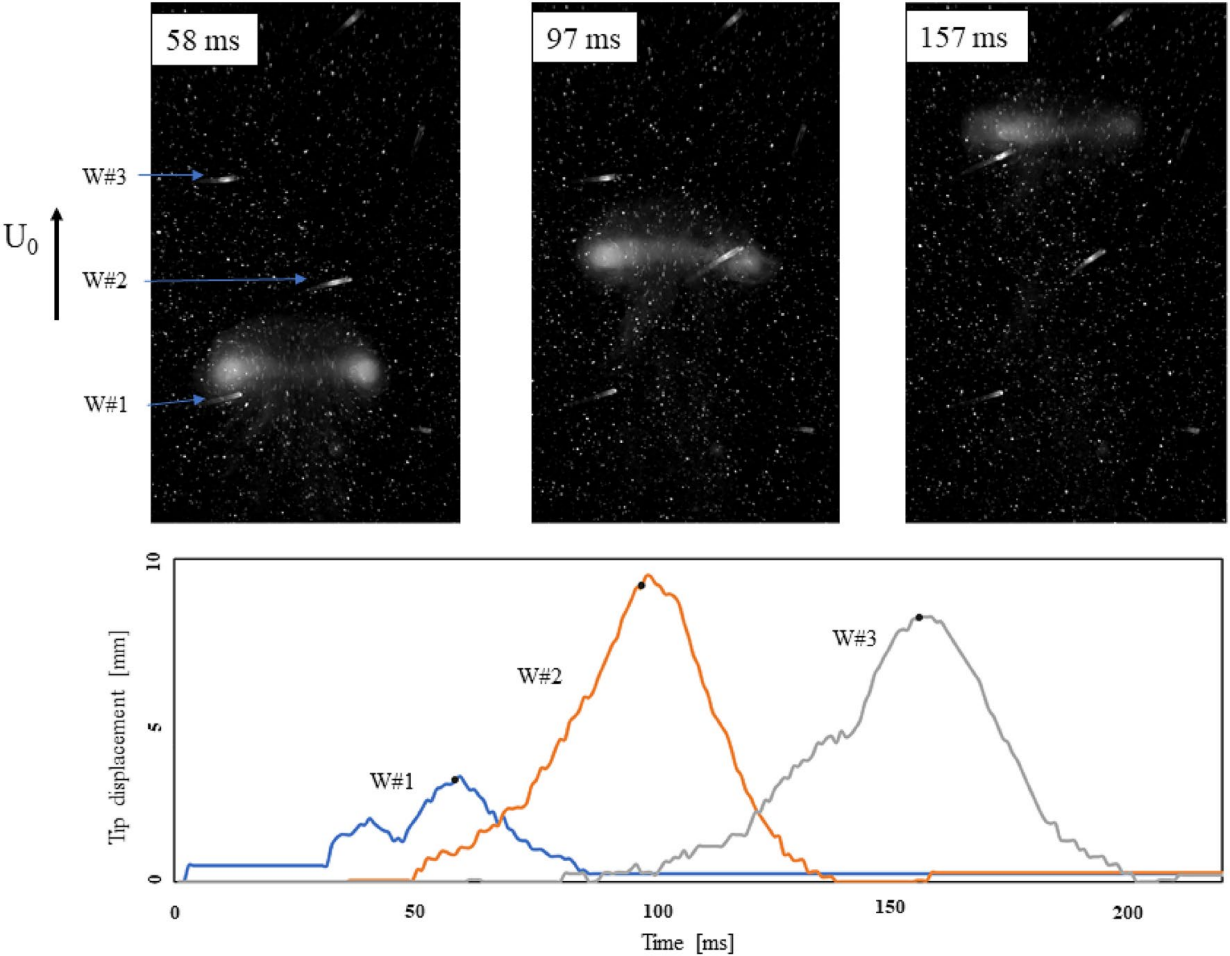
Top: Vortex ring propagating across the array. Bottom: Whisker tip tracking data matches the visualisation of the vortex ring.

Another visualisation of the interaction is given in Fig. 7, which shows a close-up view of the vortex ring interacting with a triplet of whiskers making up part of the regular array. The vortex ring is visualised by combining tracer particles and photo luminescent dye to show its shape and wake. In addition, typical whisker tip displacement profiles (relative to zero flow) are shown, to illustrate the response to the flow event passing by. The displacement magnitude includes the motion of the tips in two dimensions (longitudinal and transversal) as they interact with the vortex ring. The leading whisker W#1 is first laterally deflected by the rotation of the vortex ring before drafting into the wake and moving forward with it. This interaction results in two successive peaks in the profile of W#1, the first reflecting the rotation and the second the drafting. Successive peaks in the profiles correspond to successive deflections of whiskers W#2 and W#3. All these motions are accounted for in the CC, which solves for the best fitting TDOA.

**Figure 8:**
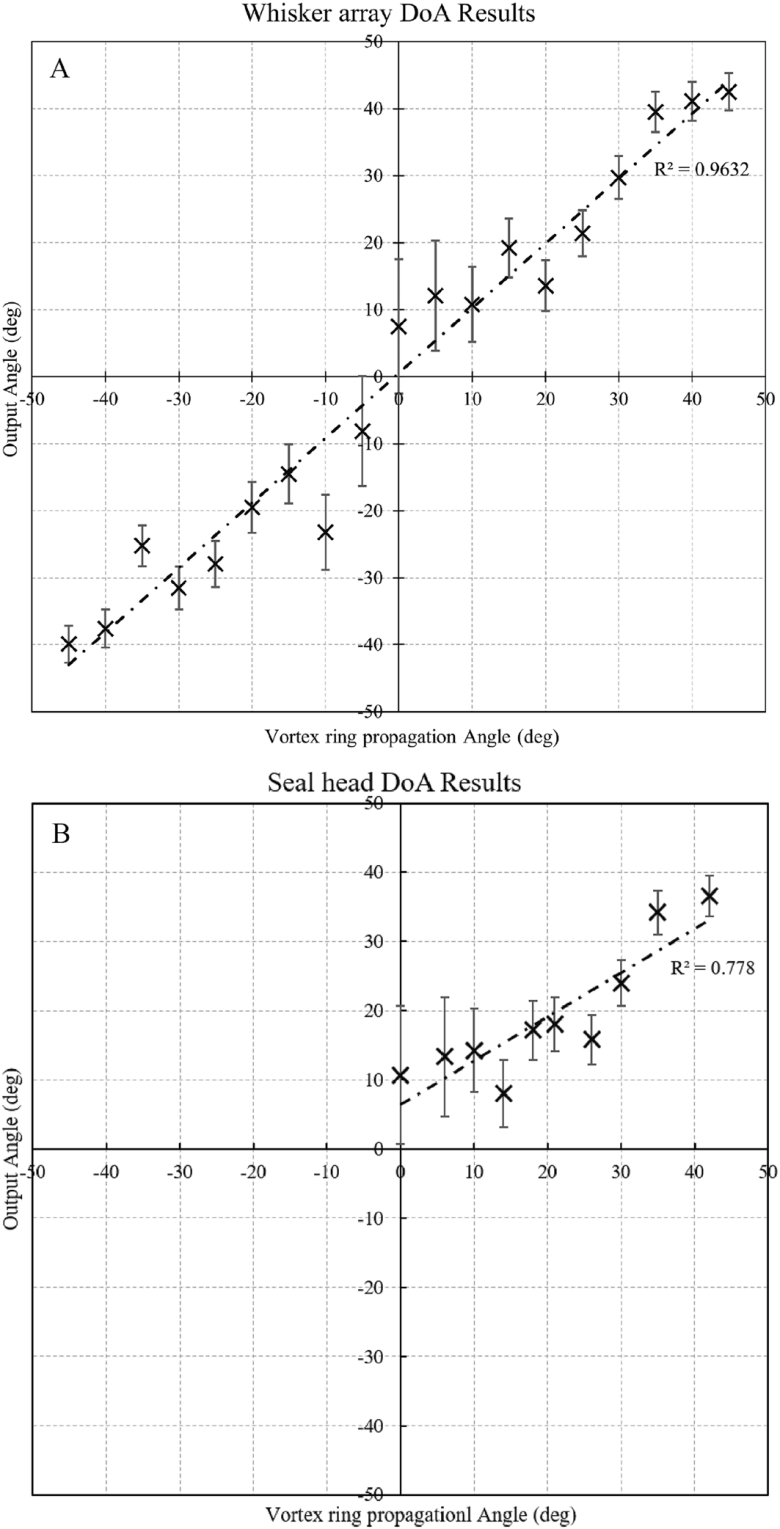
Calculated DoA angle using multilateration versus the vortex travel direction. Error bars indicate the range of DoA predictions produced from 50+ different whisker pairings per angle, from a single pulse. (**A**) regular array on the flat plat for a full 90$^\circ$ arc (Top). (**B**) array on the sea lion model tested for single-sided contralateral pad impact at angles from 0$^\circ$ to 45$^\circ$.

The processing of this data results in calculated values of the DoA angle versus the imposed vortex ring travelling direction, shown in Fig. 8. Displayed on the top are the results for the whisker array mounted on a flat plate (Fig. 8A) compared to the results for the sea lion model on the bottom (Fig. 8B). The error bars depict the uncertainty in the calculated DoA, which is dominated by poorly resolved triplets due to small angles of incidence ($\gamma$), or triplets affected by random motion in the vortex ring’s wake. On the flat plate model, all the whiskers are evenly spaced, the same length, and positioned perpendicular to the incoming vortex rings (see Fig. 4). Consequently, the position along the whisker at which the vortex impacts is roughly the same for all whiskers involved. The vortices fired with a maximum recorded vertical variation of $\pm 2.7^\circ$ to the horizontal, which even at the vortices slowest speeds, lead to a very small error of 0.11% in the calculated speed of the vortex ring. The directionality calculations based on the whisker triplets show a reasonably good trend with the imposed impact angle (linear regression with $\mathrm{R}^{2}=0.96$). This idealised setting demonstrates the effectiveness of multilateration principle applied to the whiskers when detecting the DoA of a vortex ring, evidenced by the results outside of small incidence angles.

For the sea lion model, the results shown in Fig. 8B show higher uncertainty in the calculated DoA, and the correlation of the trend (linear regression with $\mathrm{R}^{2}=0.78$) is not as strong as for the regular array. There are several contributing aspects. Firstly, the model has the whiskers retracted, with their length increasing from the nose to the downstream part of the muscle pad, approximating the natural arrangement of the vibrissae^14^. This leads to uncertainties in the position along the length of the whisker that the vortex core is impacting. This can induce an error as the calculation is based on the relative position of the tips to each other within the array, and assumes that the tip is always the first part of the whisker to be pierced by the ring. We tried to reduce this uncertainty by carefully arranging the model such that the vortex brushes past the longer whiskers, and then only considering those longer whiskers in the calculations. This reduces the number of valid triplets and increases noise. Another source of uncertainty is the possible out-of-plane component of the tip motion, which the camera is not able to detect. Therefore, the measured signal may not represent the total amplitude of tip motion other than in the case of the planar array. This effect is considered to be of lower influence.

## Discussion

We have shown via a physical demonstration, that an array of whiskers can be used as a reliable system of sensors for determining the DoA, via noise resistant GCC algorithms^12,18^, multilateration of triplets of whiskers, and using data processing methods adapted from audio and other source localisation techniques. The relevance of this study is related to the previously reported sensing capabilities of sea lions exposed to a traveling vortex ring, passing one of the vibrissal pads on their heads^10^. Consistent results of DoA detection were obtained using a similar setup with a vortex ring generator, and simplifying the system to an array of whiskers of the same length and with regular spacing. When using cross-correlation time-delay estimation with a pulsed signal, the rising or falling edge of the signal can be used as a reference mark for the time of arrival for the CC. Taking the difference in the time of arrival gives rise to the TDOA. A triplet of three sensors with two TDOA measurements are the minimum number of measurements required to calculate the DoA in a 2D plane, without knowledge of the vortices’ velocity or time of emission. This demonstration supports the idea that multilateration processing could be a method employed by the animal to detect the travel path and velocity of such disturbances.

Our results from the regular array were more consistent than from the seal head model. We explained the higher noise present in our seal head model by the fact that the whiskers in the model are retracted in the streamwise direction, which induces uncertainty in the position at which the vortex ring impacts the whiskers along their length. The calculation method assumes that the tip is the first part of the whisker which is piercing the vortex ring, which is only guaranteed to be the case with the whiskers protracted forwards. This is still open to be studied in a future experiment with a new model with protracted whiskers, supported by the observation that sea lions protract their whiskers when actively tracking^20^. It is worth considering, that studies into the minimum hydrodynamically perceivable angle of seals^10^ demonstrate that the reliability of the seals’ interpretation of the DoA drops for angles below 20$^\circ$. This aligns with measurements for the bio inspired whisker arrays in this study, with larger uncertainties at these small angles.

This aside, a regular array attached to a plate is a more practical deployment of the sensors for future applications, including autonomous underwater navigation and tracking systems. Taking multiple triplets of sensors leads to an over-determined system, which produces more accurate solutions by combining the output of all suitable triplets. In addition, machine learning or training methods may further improve the systems reliability, and reduce its sensitivity to noise. The regular pattern of the array was used as a simple layout to ensure the vortex pulse could be picked up reliably. Here, the size of the vortex ring was known, and the array was chosen such that it had sufficient spatial resolution to detect the signal with a number of triplets.

In future studies, when such an array is deployed to measure signals of unknown size or duration, and where therefore every additional valid triplet of whiskers would improve the results, one could use more specialised array layouts adapted from acoustic directionality studies^21^. These spiral array layouts use careful spacing of a multi-armed spiral to help maximise the number of useful whisker triplets that can be used reliably by avoiding very small angles of incidence with the oncoming signal or between whiskers in the triplet. For applications where the use of multiple sensors, arrays, or decentralised arrays would be beneficial, the directionality calculation functions could be expanded to utilise some form of Kalman filter to better combine readings from multiple sensors, reducing the least squared error^22^.

Applications of these techniques are manifold. Important is that such sensing would be beneficial in the near-field range to detect objects passing by, or to sense topographical structures due the wall-effect when moving along close to the ground. In such applications considerations must be made for the impact of multi-phase flows, with particles and debris typical of flows close to the ground. Moving forward we look to investigate the performance of our system in such an environment.

## Data Availability

The datasets used and/or analysed during the current study available from the corresponding author on reasonable request.
